# Exploring the unintended consequences of learning a new language at a South African university

**DOI:** 10.1371/journal.pone.0213973

**Published:** 2019-03-20

**Authors:** Mike Murray

**Affiliations:** University of KwaZulu-Natal, School of Mathematics, Statistics and Computer Science, Westville Campus, Durban, KwaZulu-Natal, South Africa; University of California Santa Barbara, UNITED STATES

## Abstract

The University of KwaZulu-Natal has introduced a new language policy requiring all students who have not done Zulu as a first (or second) language at school to complete a six-month course in Zulu as part of their undergraduate curriculum. Given that English is the medium of instruction that is being used for all their other courses, the time that these students have had to spend learning this new language may be adversely affecting the marks that they could be getting for their other courses. Having to do a course in Zulu is also preventing them from being able to take another course in their chosen area of study. If one were able to randomly assign students to the group who have to take the Zulu course, then any difference in performance between the two groups could be directly attributed to the new language policy that is being introduced at the university. Given the context of this paper, however, such a random allocation is not possible. Students who have not done Zulu at school have to eventually complete a course in Zulu before graduating. If this cohort differs (in any other way) from those who do not have to take this course, then any observed difference in overall performance between the two groups may be a result of these group differences rather than the new rule that has been implemented.

**Methodology:**

This paper uses regression adjustment and entropy matching to achieve an appropriate balance between the two groups. Having achieved this balance any difference in the overall performance between the two groups can then be attributed directly to the new language policy that has been implemented. Our results indicate, after matching, that a significant difference in performance between the two groups occurs with Black African Zulu home language speakers in particular not performing as well as students from the other race and language groups. The data came from the University of KwaZulu-Natal and the study has been approved by the University Ethics committee and Research committee.

**Results and conclusions:**

Using a weighted mean of marks to measure performance, the ATT estimates that result indicate that students in the treated group would all perform significantly better had they been allowed instead to enrol in the non-treated group. Furthermore, Zulu home language speakers, who are not actually forced to take this course, are significantly underperforming whether they have chosen to take this course in Zulu or not. Surprisingly, their underperformance is worse in the treated group. Because one would expect them to be scoring a higher mark for Zulu in this treated group, forfeiting the chance to take another course in their chosen area of study is clearly affecting the type of mark they could be getting for their other courses had they chosen to remain in the non-treated group. With English being the medium of instruction at this university, should the university not also consider introducing a compulsory course in English for these Zulu home language speakers? Females are doing better than males in both groups. The effect being stronger in the treated group suggests that females appear to cope better with the learning of a new language. Significant college effects are also being observed, suggesting that this new language rule possibly needs to be adjusted for the college a student wants to study in.

## Introduction

When students enrol for a degree, should their curriculum focus solely on the teaching of subject material that relates directly to their chosen area of study or should it also include other subjects that will help to broaden the educational background of a prospective student. In South Africa, high school students have to study two languages, one at a first language level and the other at a second language level. Most students opt to study English as a first language taking Zulu or Afrikaans as a second language. Because the University of KwaZulu-Natal is located in an area of South Africa where Zulu is spoken by more than 80% of the local population, being able to understand or speak that language will clearly have many important socio- and economic benefits. This line of thought has prompted the University of KwaZulu-Natal (UKZN) to introduce a language policy in 2014 requiring all students who have not done Zulu as a first (or second) language at school to complete a six-month course in Zulu as part of their undergraduate curriculum. Being forced to take a course in Zulu however effectively prevents them from being able to take another course in their chosen field of study. Will the time that they have had to spend learning this new language adversely affect the marks that they could be getting for their other subjects. Using a weighted mean of marks to measure performance, can a significant difference in weighted means between the group who have to take Zulu and the group who do not actually represent a treatment effect that one can associate with the new language policy that is being introduced at UKZN? For example, if students in the one group are predominantly female whereas those in the other group are predominantly male, then one could just as easily argue that the difference in overall performance that one is observing between the two groups is being caused by gender rather than the new rule that has been implemented.

Randomisation is a tool that is often used to isolate the confounding effect of a background variable (such as gender) from that of a particular treatment assignment mechanism. In the context of this paper treatment refers to the cohort of students who have chosen to take the Zulu course as part of their degree in 2014. Being able to randomly assign students to a particular group helps to create a treated and untreated group with similar (or matched) background characteristics. Any difference in performance between the two groups can now be attributed directly to the new language policy because potential differences attributable to these other background characteristics would have been eliminated by the random assignment process. Unfortunately, given the context of this paper, a random assignment of students to a particular group is not possible. Zulu home language speakers have a choice to either enrol for this course or to take another course in their chosen field of study. Students who have not done Zulu at school however must eventually complete a six month course in Zulu at UKZN.

To address this problem we have used regression adjustment and entropy matching to help achieve an appropriate balance between the two groups. Having achieved this balance, any difference in the overall performance between the two groups can then be attributed directly to the new language policy that has been implemented at the university.

More specifically, in this paper, we will be wanting to consider the effect that this new language policy is having on three different cohorts of students; namely

- Black African students who speak Zulu as a home language
- Black African students who speak do not speak Zulu as a home language, and
- Non-Black African (predominantly European and Indian) students who make up the rest of the student population at UKZN.

Enrolment figures for each of these student groups are given in Table 1.

**Table 1:** Student enrolment at UKZN in 2014.

2014 Intake	Zulu home language speaker	Other home language speaker
Black African	26,322	5,324
Not Black African	10	16,877

For Zulu home language speakers the benefit associated with doing a soft option course in Zulu will have to be compared with the benefit that would be derived from being able to take another course in one’s chosen field of study. For Black African students who speak another African language, not being able to take an extra course in their chosen field of study may impact negatively on the results they could be achieving for their other subjects had the new language rule not been in place. Many African languages however have a common root. Consequently having to complete a course in Zulu may not be as difficult a learning process for them as would be the case for a European or Indian student who would also have to eventually complete a course in Zulu at UKZN.

### Should the Zulu speaking students be asked to complete a course in English?

The benefits associated with being able to learn a new concept in one’s mother tongue have been well documented in the literature [1–10]. Because English is the medium of instruction that is being used at UKZN, should the Zulu speaking students not also be asked to take a similar type of bridging course in English to help them better understand some of the concepts that will be taught in the other courses that they will be taking? Local studies [5–6] have found that a culture of rote learning is widely prevalent amongst many second language learners in South African universities. Asking a non-Zulu home language speaker to complete a course in Zulu has a clearly defined socio-economic benefit for them. Asking a Zulu home language speaker to complete a similar course in English may help them to overcome a significant language barrier that is being hidden behind a culture of rote learning.

## Methodology

Let *T*_*i*_ denote a 0/1 treatment indicator variable that is being set equal to one if student *i* chooses to take Zulu in 2014 and zero otherwise. Let *Y*_*i*_ represent a weighted mean mark for all the exams that student *i* writes in 2014. Observations on *Y*_*i*_ and *T*_*i*_ can then be used to compute the following expression

$$\sum_{i=1}^{n}T_{i}Y_{i}/\sum_{i=1}^{n}T_{i}‑\sum_{i=1}^{n}\left(1-T_{i}\right)Y_{i}/\sum_{i=1}^{n}\left(1-T_{i}\right)$$ (1)

which essentially subtracts a sample mean of outcomes on *Y*_*i*_ for students in the non-treated group from a sample mean of outcomes on *Y*_*i*_ for students in the treated group.

If the allocation of students to a treated or untreated group is being done in a random manner then (1) provides one with an estimate for an effect that can be attributed directly to the treatment variable alone.

Student allocation to a treated or non-treated group however is not being done on a random basis. Whereas Zulu speaking students have the option to enrol for this course, students who have not done Zulu at school have to eventually complete this course as part of their degree structure. Ideally one would like to be able to observe a weighted mean mark *Y*_1*i*_ that student *i* obtains for all their subjects if they took Zulu in 2014 and also a weighted man mark *Y*_0*i*_ had that same student chosen not to take Zulu in 2014.

The following expression could then provide one with a sample based estimate of the effect on academic performance that the new language policy is having

$$\sum_{i=1}^{n}(Y_{1i}-Y_{0i})/n$$ (2)

Unfortunately, the above expression can never be calculated because only one of the outcomes *Y*_1*i*_ and *Y*_0*i*_ can actually be observed once a student has made a decision *T*_*i*_ to take (or not to take) Zulu in 2014. Fortunately, given some additional assumptions

$$\mathrm{ATE}=E\left(Y_{1i}-Y_{0i}\right)$$

and

$$\mathrm{ATT}=E\left(Y_{1i}-Y_{0i}|T_{i}=1\right)$$

are two causal effects that one may be able to estimate using the data *Y*_*i*_ that one is able to observe. In the context of this paper, ATE represents an average treatment effect on *Y*_*i*_ that one can associate with every student who enrols for a degree in UKZN during 2014 whereas ATT represents an average treatment effect that applies only to those students who have chosen to take Zulu as part of their curriculum in 2014. A negative value for ATT would indicate that had these students in this treated group not been required to take a course in Zulu then they would have done better in their other courses.

### Estimating ATT using a regression adjustment approach

A treatment assignment mechanism is said to be strongly ignorable if *T*_*i*_ and (*Y*_0*i*_,*Y*_1*i*_) become independent after conditioning on a set of background variables X.

Such an assumption ensures that we have

$$E\left(Y_{i}|X,T=1\right)=E(T_{i}Y_{1i}+\left(1-T_{i}Y_{0i}|X,T=1\right)=E\left(Y_{1i}|X\right)$$

which allows E(*Y*_1*i*_|*X*) to be conditionally estimated from one’s observed data using only those outcomes on *Y*_*i*_ that have come from the treated population. Similarly

$$E\left(Y_{i}|X,T=0\right)=E(T_{i}Y_{1i}+\left(1-T_{i}Y_{0i}|X,T=0\right)=E\left(Y_{0i}|X\right)$$

which allows E(*Y*_0*i*_|*X*) also to be conditionally estimated from one’s observed data using only outcomes on *Y*_*i*_ that have come from the untreated population. These results underpin a regression adjustment method that can then be used to estimate ATT and thus determine whether or not the new language policy is adversely affecting the performance of students who are having to complete a course in Zulu as part of their degree. Details of the estimation method are given in an appendix to this paper.

### Estimating ATT by appropriate matching on a entropy score

Matching is another nonparametric method that one could consider using to adjust for the confounding effect that a set of background variables X may be having on our outcome variable Y. Matching attempts to mimic a randomized experiment by adjusting (through the reweighting or discarding of units in the control group) the empirical distribution associated with the background variables X in the control group so that it closely `matches’ that of the covariates X in the treatment group. With an appropriate balance having been achieved, controlling further for X becomes unnecessary since it is now unrelated to the treatment variable T and thus a difference in the weighted means on the matched data can be used to estimate the causal effect. For example,

$$\hat{ATT}=N_{T}{}^{-1}\sum_{\left\{i:T_{i}=1\right\}}^{n}\left(Y_{i}-\sum_{\left\{j:T_{j}=0\right\}}^{n}w_{ij}Y_{j}\right)$$ (3)

can be used to estimate ATT with *N*_*T*_ representing the number of observations in the treated group that have been collected and *w*_*ij*_ the weight that is being assigned to a non-treated outcome *j* based on an appropriate match with an observation *i* in the treated group.

Typically the matching of an observation in the treated group with one or more observations in the control group requires the calculation of a propensity score or an appropriately chosen distance measure [11–20]. One then has to check whether the balance that is being achieved is adequate and perform another rematch (or discarding of units) if this is not the case. Unfortunately, with each iteration, there is no guarantee that an improvement in balance will be achieved. In addition, ATT estimation can often perform poorly [17] particularly when there is insufficient overlap in the covariate distributions associated with the treated and control groups. To help overcome these problems, Hainmueller [20] has developed a reweighting scheme for the non-treated observations that asks the user to pre-specify a desired level of covariate balance that they want to achieve between the covariates in the treated and control group. For example, one may want a balance (between the treated and untreated populations) on the mean, variance and third moment of a chosen set of background variables X to be achieved. Subject to these balance conditions being satisfied, a set of weights *w*_*ij*_ for the non-treated observations in (3) are then derived that minimize an appropriately chosen Kullback-Leibler entropy score. Because the balancing constraints are being directly built into the reweighting scheme that is being implemented, a check for balance after each reweighting no longer becomes necessary. The weights that result from this entropy balancing can then be used as inputs for estimating a desired treatment effect. For example, they could be used to appropriately reweight a difference in means estimator for ATT or to fit a weighted least squares model to one’s chosen outcome variable Y using the treatment variable T and the confounding variables X as regressor variables in one’s analysis [12].

## Results

The academic performance of 48,062 students enrolled for a degree at UKZN in 2014 formed the basis for this study. The data came from the University of KwaZulu-Natal and the study has been approved by that universities Ethics committee and Research committee. The year 2014 was chosen because that was the year in which the new language policy was first implemented. An appropriate measure for academic performance was obtained by adding up the percentage mark obtained for each subject taken in 2014 and dividing that by the total number of subjects written over the year. Students take a total of eight courses over the year. These courses are split into two half year semesters with the Zulu course being able to be taken in either semester. If Zulu is taken then it replaces one of the other courses that could be taken as part of their degree structure.

A summary of results obtained for the treated and non-treated groups is given in Table 2.

**Table 2:** Weighted mean mark being recorded for 2014.

	Number of students	Mean	Standard deviation
Treated group (Took Zulu in 2014)	5819	54.56	14.59
Non-treated group (Did not take Zulu in 2014)	42,243	53.52	14.42
Total number of Students	48,062	53.64	14.44

ATE = 54.56–53.52 = 1.04; t-test:5.771

A two-sample t-test for a difference in means between the marks being recorded by the treated and non-treated groups respectively produced a t- value of 5.771 (p-value = 0.002) indicating that students in the treated group perform marginally better than those in the non-treated group. Before one can conclude that the new language policy is actually helping them to perform better in all their other studies one needs to make sure that there are no other background variables that may be masking the true effect that the taking of a course in Zulu may be having on the mean marks that students are recording in the treated and control groups.

Table 3 lists the background variables that we have chosen to include in our analysis. In South Africa, schools have been ranked into quintiles based on the resources that they have at their disposal with the quintile 1 schools being the poorest and the quintile 5 schools the richest. The variable Quint12 that appears in Table 2 refers to a student who has come from a poor schooling background. When a student writes their final school leaving exams the results that they obtain are often summarised in the form of an ordinal point score for each subject. The higher the point score, the better the result that they have achieved for that particular subject. These point scores are then added up producing a Matric Point score which we have called MatPts in Table 3.

**Table 3:** Confounding variables that have been included in the analysis.

Variable name	Abbreviation	Type of variable	Assignment definition
Female	Female	0/1 indicator	1 = female student 0 = male student
Black African	Black African	0/1 indicator	1 = Black African student 0 = from another race group
Quintile 1 or 2 school background	Quint12	0/1 indicator	1 = went to a quintile 1 or 2 school 0 = went to a more privileged quintile 3,4 or 5 school
Matric Point score	MatPts	integer valued score	Matric point score comprising a weighted mean of scores for one’s school leaving subjects.
College: Agriculture, Engineering & Science (AES)	AES	0/1 indicator	1 = AES student 0 = from another College
Health Sciences	Health	0/1 indicator	1 = Health Sciences student 0 = from another college
Humanities	Human	0/1 indicator	1 = Humanities student 0 = from other college
Law & Management	LawMan	0/1 indicator	1 = Law & Management student 0 = from another college
Zulu home language speaker	Afzulu	0/1 indicator	1 = home language is Zulu 0 = home language not Zulu

The academic structure at UKZN has been divided into four colleges. Binary indicator variables will be used to model the effect of these colleges in the results that follow. To avoid identification issues, however, only the indicator variables for the first three colleges will be used with an effect for the College of Law and Management Studies forming part of the intercept term in each model structure.

To check for a possible imbalance in the distribution of background variables between the treated and non-treated groups, appropriate proportions and variances were calculated for each of the background variable being given in Table 3. A test for a difference in proportions between the treated and non treated groups using the following asymptotically normally distributed test statistic

$$Z=\frac{{\hat{p}}_{1}-{\hat{p}}_{2}}{\sqrt{\hat{p}}(1-\hat{p)[\frac{1}{n_{1}}+\frac{1}{n_{2}}]}}\;\hat{p}=\frac{n_{1}{\hat{p}}_{1}+n_{2}{\hat{p}}_{2}}{n_{1}+n_{2}}$$

was also done. The results in Table 4 indicate a strong imbalance in the covariate distributions between the treated and control groups for all covariates except possibly the covariate Afzulu indicating whether the student is Zulu home language speaker or not.

**Table 4:** Testing for a difference in proportions between the treated and non-treated populations.

Covariate	Estimated Proportion (Treated group)	Variance	Estimated proportion (Non-treated group)	Variance	Test statistic for a difference in proportions between the two groups (p-values)
Female	0.720	0.201	0.569	0.244	-22.106*
Black African	0.596	0.241	0.656	0.226	9.089*
AES	0.092	0.083	0.293	0.207	32.355*
Health	0.453	0.248	0.407	0.241	-6.787*
Humanities	0.082	0.075	0.262	0.193	30.134*
Afzulu	0.522	0.249	0.55	0.248	3.638(0.00013)
Quint12	0.128	0.111	0.144	0.123	3.286(0.00051)

*denotes significant at 5% level and denotes p-values of order less less than 10^−10^.

### Analysis based on a regression adjustment

Estimates for the mean treatment effects that are given in Table 4 indicate that having to take a course in Zulu is actually lowering the overall weighted mean mark that one can expect to record by more than 2 percentage points with this effect being stronger amongst the treated subpopulation who have actually chosen to take Zulu in 2014. This contradicts an earlier conclusion that we would have been made (see Table 2) had we chosen to make no adjustment for potential confounders in our analysis.

Ordinary least squares applied to model structure (A3) that appears in the supporting appendix produced the results that are given in Table 5 and Table 6. The Stata code that was used to generate these results also appears in the supporting information section of this paper.

**Table 5:** Estimated average treatment effects using weighted mean mark as a response variable.

Average Treatment Effect	Estimate	95% Confidence interval
ATE	-2.094	[-2.585;-1.602]
ATT	-2.509	[-2.944;-2.074]

**Table 6:** Parameter estimates associated with academic performance at UKZN in 2014.

Covariate	Estimate (Non-treated group)	Standard Error	Estimate (Treated group)	Standard Error
Female	0.338*	0.141	2.591*	0.428
Black African	0.457	0.272	0.583	0.832
AES	-2.767*	0.178	-5.295*	0.758
Humanities	1.383*	0.177	-3.194*	0.636
Health	7.781*	0.362	3.915*	0.582
Afzulu	-4.116*	0.251	-6.167*	0.822
Quint12	0.511*	0.205	1.179*	0.539
MatPts	0.195*	0.008	0.309*	0.027
Intercept	49.169*	0.334	46.102*	1.120

*denotes significant at 5% level

Females appear to perform significantly better than males with the effect being stronger in the treated subpopulation. This outcome may be reinforcing a notion that females are better at learning a new language bearing in mind that this is an effect that is being recorded after an adjustment for all the other included variables in the model has been made.

Not unexpectedly, students with a higher Matric point score perform better than those with a lower point score with the effect being slightly more stronger in the treated subpopulation. Interestingly enough, race (being a Black African) becomes insignificant once an adjustment for being a Zulu home language speaker (Afzulu) has been made.

Within both groups, Zulu home language speakers significantly underperform when compared with those who speak another home language with the effect being stronger in the treated group. This is surprising because Zulu home language speakers in the treated group would be taking a course in Zulu in which they would be expected to perform well. Perhaps the extra course in Zulu that is being offered at UKZN is having an unintended consequence in that it is providing Zulu home language speakers with a `soft-option’ to register for when in fact they would benefit more by remaining in the non-treated group where an extra course in their chosen field of study could be taken.

Because Zulu home language speakers are strongly underperforming in both groups, these results could indicate that they are battling to cope with English that is being used as a medium of instruction for their other subjects at UKZN. Should UKZN not also be offering a bridging course in English for these Zulu home language speakers?

The college effects are all very significant noting that students in the College of Law and Management Studies are forming a baseline college with which to make a comparison. Students in the College of Agriculture, Engineering & Science (AES) do not do as well as their counterparts in this baseline college based category. Students in the College of Health Sciences however do much better than students in the College of Law and Management Studies. In all these colleges, however, students in the non-treated groups perform significantly better than students in the treated groups.

### Matching based on a entropy score

To help support the results that have been obtained in Table 5 using a regression adjustment procedure, Hainmueller’s entropy balancing procedure was run, using a Stata package called ebalance [21] to create a set of matching weights that could then be used as inputs in a weighted regression model for the subsequent estimation of a treatment effect. Results from this analysis are given in Table 7.

**Table 7:** Parameter estimates obtained for the weighted mean mark being recorded in 2014 using the entropy balancing method of Hainmueller.

Covariate	Estimate	Standard Error	95% Confidence Interval
Treatment	-2.731*	0.225	[-3.173;-2.289]
Female	1.697*	0.255	[1.196;2.198]
Black African	0.154	0.514	[-0.852;1.162]
AES	-4.398*	0.393	[-4.758;-3,218]
Humanities	-0.445	0.329	[-1.091; 0.199]
Health	6.075*	0.366	[5.357; 6.7931]
Afzulu	-5.719*	0.480	[-6.660; -4.777]
quint12	0.647*	0.326	[0.008; 1.288]
MatPts	0.304*	0.018	[0.267;0.341]
Intercept	47.537*	0.739	[46.087;48.987]

*denotes significant at 5% level of significance.

The estimate -2.731 associated with Treatment represents an ATT effect that is of a similar order to the estimate that we obtained for ATT in Table 5. Parameter estimates associated with the other included covariates also show similar effects to those reported earlier in the regression adjustment based analysis. Zulu speaking students significantly underperform, females do better than males and college effects are important with students in the College of Agriculture, Engineering and Science (AES) not doing as well students in the other colleges.

## Conclusions

This paper is wanting to explore the possibly unintended consequences arising from a decision to make Zulu a compulsory subject for students who have not learnt Zulu at school. Whereas the advantages associated with learning a new language can easily be supported from a socio-economic point of view, from an academic performance point of view, the ATT estimates that we have obtained suggest that these students would be obtaining higher marks for their other subjects if they weren’t forced to complete this course in Zulu. One should,not, however, ignore the long term social benefits that will obviously accrue later in life from being able to understand a local language. Consequently this paper is not advocating the discontinuation of this rule. Rather, this paper is wanting to quantify the value of this effect noting that one’s average mark for the entire year decreases by 3% if one includes Zulu in one’s subject choice for the year.

For Zulu home language speakers who do not actually have to take this course, the temptation to enrol for such a course is preventing them from being able to take another course in their chosen field of study. The ATT estimates that we have obtained suggest that by choosing to take what for them would be a soft option course in Zulu, they too are also not performing as well as they could have in their other subjects had they chosen to remain in the non-treated group. Because Zulu home language speakers are also underperforming significantly in the non- treatment group (see Table 6) perhaps the source of this problem relates to a lack of proficiency in English which is being used as a medium of instruction for all their other courses at UKZN. To mitigate against this unintended consequence, perhaps the language policy at this university needs to be adjusted so as to include a compulsory bridging course in English for Zulu home language speakers to help them overcome the language barrier that they are being confronted with.

## Supporting information

S1 Dataset Dataset containing weighted mean marks and university specific demographic factors. (DTA)

S1 Stata code Program code that has been used to generate the results in this paper. (DOCX)

S1 Appendix Derivation of the formulae that have been used to estimated the treatment effects in this paper. (DOCX)

## References

[1] Taylor, S and Coetzee, M. Estimating the impact of language of instruction in South African primary schools: A fixed effects approach Stellenbosch Working paper Series N0 WP21/2013. Available at http://www.ekon.sun.ac.za/wpapers/2013

[2] Pandor, N. Language Issues and Challenges (opening address at the Language Policy Implementation in HEIs Conference, Pretoria, 5 October 2006. Available at http://www.education.gov.za/dynamic/dynamic.aspx?pageid=306&id=2290.

[3] Schuring G. K. Language and Education in South Africa: A policy study. Pretoria: Human Sciences Research Council, 1993.

[4] Vygotsky L. S. Mind in society: the development of higher psychological processes. Cambridge, MA: Harvard University Press, 1978.

[5] Johnstone A. and Selepeng D. A language problem revisited. Chemistry Education: Research and Practice in Europe, 2(1), 19–29, 2001.

[6] Bird E. and Welford G. The effect of language on the performance of second-language students in science examinations. International Journal of Science Education, 1995: 17(3), 389–397.

[7] Cassels J. and Johnstone A. Meaning of words and teaching of chemistry. Education in Chemistry, 1983:20 (1), 10–11.

[8] Cassels J. and Johnstone A. Words that matter in science. London: Royal Society of Chemistry, 1985.

[9] Pollnick M. and Rutherford M. The use of a conceptual change model and mixed language strategy for remediating misconceptions in air pressure. International Journal of Science Education, 1993:15, 363–381

[10] Howe M. Introduction to human memory: A psychological approach. New York: Harper and Powe, 1970.

[11] Stuart E.A. Matching methods for causal inference: A review and a look forward. Statistical Science,2010: 25(1),1–21. 10.1214/09-STS313 20871802 PMC2943670

[12] Imbens G.W. Matching methods in practice: Three Examples. Journal of Human Resources 2015: 50,2,373–419.

[13] Abadie A. and Imbens GW. Large sample properties of matching estimators for average treatment effects. Econometrica, 2006: 74(1):235–267.

[14] Abadie A. and Imbens GW. Bias corrected matching estimators for average treatment effects. Forthcoming in the Journal of Educational and Behavioral Statistics.2009 Available at www.hks.harvard.edu/fs/aabadie/bcm.pdf.

[15] Abadie A and Imbens GW. Working Paper 15301. National Bureau of Economic Research; Cambridge, MA: Matching on the estimated propensity score, 2009.

[16] Ho DE, Imai K, King G and Stuart EA. Matching as nonparametric preprocessing for reducing model dependence in parametric causal inference. Political Analysis, 2007:15(3):199–236.

[17] Glazerman S, Levy DM, Myers D. Nonexperimental versus experimental estimates of earnings impacts. The Annals of the American Academy of Political and Social Science, 2003:589:63.

[18] Dehejia RH and Wahba S. Causal effects in nonexperimental studies: Re-evaluating the evaluation of training programs. Journal of the American Statistical Association, 1999:94:1053–62.

[19] Dehejia RH and Wahba S. Propensity score matching methods for non-experimental causal studies. Review of Economics and Statistics, 2002: 84:151–161.

[20] Hainmueller J. Entropy Balancing for Causal Effects: A Multivariate Reweighting Method to Produce Balanced Samples in Observational Studies. Political Analysis 2012: 20 (1): 25–46.

[21] Hainmueller J. and Xu Y.x ebalance: A Stata package for entropy balancing. Journal of Statistical Software. 2103: 54 (7), 1.

