# Supplementary material for: Exploring the unintended consequences of learning a new language at a South African university
Source: PLoS One. 2019 Mar 20;14(3):e0213973. doi: 10.1371/journal.pone.0213973 (PMC6426258; doi:10.1371/journal.pone.0213973)
Supplement: S1 Stata code — (DOCX) [file pone.0213973.s002.docx]

**S1 Stata code.**

. use "zulustataver12.dta"

. sum WavYear if treated==1

. prtest female, by (treated)

. prtest BlackAfrican, by (treated)

. prtest AES, by (treated)

. prtest Humanities, by (treated)

. prtest HealthSciences, by (treated)

. prtest ManagementStudies, by (treated)

. prtest afzulu, by (treated)

. prtest quint12, by (treated)

. teffects ra (WavYear female BlackAfrican MatPts AES HealthSciences Humanities afzulu quint12) (treated)

. teffects ra (WavYear female BlackAfrican MatPts AES HealthSciences Humanities afzulu quint12) (treated) , atet

. teffects ra (WavYear female BlackAfrican MatPts AES HealthSciences Humanities afzulu quint12) (treated) , aequations

.ssc install ebalance

.ebalance zuluniv2 sex1 race1 MatPts college1 college2 college3 afzulu quint12, targets(1)

. svyset [pweight=_webal]

. svy: reg WavYear zuluniv2 sex1 race1 MatPts college1 college2 college3 afzulu quint12
