# Supplementary material for: Exploring the unintended consequences of learning a new language at a South African university
Source: PLoS One. 2019 Mar 20;14(3):e0213973. doi: 10.1371/journal.pone.0213973 (PMC6426258; doi:10.1371/journal.pone.0213973)
Supplement: S1 Appendix — (DOCX) [file pone.0213973.s003.docx]

**S1 Appendix.**

Assume that the potential outcomes $Y_{0i}$and $Y_{1i}$are being generated by the following two regression model structures

$Y_{0i}=\alpha_{0}$ +$X_{i}$ $\beta_{0}$ + $e_{0i}$ (A1)

and

$Y_{1i}=\alpha_{1}$ + ${X_{i}\beta}_{1}$ + $e_{1i}$ (A2)

with $e_{0i}$and $e_{1i}$representing two error terms both having a zero mean. Substituting (A1) and (A2) into the following expression linking our observed data to the potential outcome variables $Y_{0i}$ and $Y_{1i}$, viz.

$Y_{i}=T_{i}Y_{1i}$+$(1-T_{i}{)Y}_{0i}$

produces

$Y_{i}=\alpha_{0}+$ ${(\alpha}_{1}-\alpha_{0})T_{i}+$ ${X_{i}\beta}_{0}+T_{i}$ ${X_{i}(\beta}_{1}-\beta_{0})$+$\varepsilon_{i}$ (A3)

with

$${\varepsilon_{i}=e}_{0i}+ {T_{i}(e}_{1i}-e_{0i})$$

representing an error term for this observed outcome model. Conditioning on $T_{i}$ and $X_{i}$

${E[Y}_{i}| T_{i}$ , $X_{i}]= \alpha_{0}+$ ${[\alpha}_{1}-\alpha_{0}+{\mu_{X} (\beta}_{1}-\beta_{0})]T_{i}+$ ${X_{i}\beta}_{0}$

$+T_{i}($ ${X_{i}-\mu_{X} )[\beta}_{1}-\beta_{0}]$+$E[\varepsilon_{i}$ $| T_{i}$ , $X_{i}]$ (A4)

with $\mu_{X}=E(X)$ denoting a mean response vector for one’s fully observed set of confounding variables X. Under a strong ignorability condition it follows from (A4) that

ATE(X)≡ ${E[Y}_{1i}|$ $X_{i}]- {E[Y}_{0i}|$ $X_{i}]{=E[Y}_{i}| T_{i}=1$ , $X_{i}]- {E[Y}_{i}| T_{i}=0$ , $X_{i}]$

$={(\alpha}_{1}-\alpha_{0}) {+ X(\beta}_{1}-\beta_{0}$)

Thus an estimate

$\hat{\tau}_{ate,reg}$ =${(\hat{\alpha}}_{1}-\hat{\alpha}_{0})+$ ${\bar{x} [\hat{\beta}}_{1}-\hat{\beta}_{0}]$

for

ATE=$E_{X}\{ATE$(X)}= ${(\alpha}_{1}-\alpha_{0}) {+ \mu_{X}(\beta}_{1}-\beta_{0})$

can be obtained by applying OLS estimation on the following regression model structure that is being implied by (A4); viz.

${Y_{i}=\alpha_{0}+ [\alpha}_{1}-\alpha_{0}+{\mu_{X} (\beta}_{1}-\beta_{0})]T_{i}+$ ${X_{i}\beta}_{0}+T_{i}($ ${X_{i}-\mu_{X} )[\beta}_{1}-\beta_{0}]$ $+ \varepsilon_{i}$ (7)

Similarly,

$\hat{\tau}_{att,reg=}\hat{\tau}_{ate,reg}+\frac{\boldsymbol{1}}{\sum_{\boldsymbol{i}}^{\boldsymbol{N}} \boldsymbol{T}_{\boldsymbol{i}}}$ $\sum_{\boldsymbol{i}}^{\boldsymbol{N}} {\boldsymbol{T}_{\boldsymbol{i}}\boldsymbol{(}\boldsymbol{X}_{\boldsymbol{i}}\boldsymbol{-}\bar{\boldsymbol{x}}\boldsymbol{)(}\hat{\boldsymbol{\beta}}}_{\boldsymbol{1}}\boldsymbol{-}{\hat{\boldsymbol{\beta}}}_{\boldsymbol{0}}\boldsymbol{)}$

provides one with a consistent estimate for

ATT = $E_{X{|T}_{i}=1}\left\{ ATE\left( X \right) \right\}$

=$E_{X{|T}_{i}=1}\{{(\alpha}_{1}-\alpha_{0}) {+( X- \mu_{X})(\beta}_{1}-\beta_{0}){+ \mu_{X}(\beta}_{1}-\beta_{0})\}$

=ATE+$E_{X}[$ ${X-\mu_{X}|T=1 ][\beta}_{1}-\beta_{0}]$

If we are willing to assume that $\beta_{1}=\beta_{0}$ and $E(\varepsilon_{i}|T_{i}, X_{i})$=0, then

ATE= $\alpha_{1}-\alpha_{0}$

represents a homogenous mean treatment effect which can be consistently estimate by applying ordinary least squares on the following regression model

$Y_{i}=\alpha_{0}+$ ${(\alpha}_{1}-\alpha_{0})T_{i}+$ ${X_{i}\beta}_{0}+ \varepsilon_{i}$
